# Prevention of type 2 diabetes in urban American Indian/Alaskan Native communities: The Life in BALANCE pilot study

**DOI:** 10.4236/jdm.2013.34028

**Published:** 2013-11-01

**Authors:** Daniel C. Benyshek, Michelle Chino, Carolee Dodge-Francis, Toricellas O. Begay, Hongbin Jin, Celeste Giordano

**Affiliations:** 1 Department of Anthropology, University of Nevada Las Vegas, Las Vegas, USA; 2 School of Community Health Sciences, University of Nevada Las Vegas, Las Vegas, USA

**Keywords:** Type 2 Diabetes, Community-Based Participatory Research, Prevention, American Indian/Alaskan Native, Urban

## Abstract

**Objective:**

The Life in BALANCE (LIB) study is a pilot translational study modeling the Diabetes Prevention Program (DPP) intensive lifestyle coaching intervention among an underserved, high-risk population: American Indians/Alaska Natives (AI/ANs) living in a large urban setting (Las Vegas, Nevada).

**Research Design and Methods:**

A total of 22 overweight/obese AI/ANs (age, 39.6 ± 10.4 years; BMI, 34.1 ± 6.3 kg/m^2^) at increased risk for developing type 2 diabetes (HbA1c > 5.4 (36 mmol/mol) < 6.4 percent (46 mmol/mol) participated in the program between April and December, 2011. Study participants completed a 16 week intensive lifestyle coaching intervention. In addition to obtaining qualitative data regarding opportunities and challenges of applying the lifestyle intervention for AI/AN participants in an urban setting, clinical data, including BMI, waist circumference, blood pressure, fasting blood glucose, and blood lipids (HDL, LDL and Triglycerides), were collected.

**Results:**

Only 12 of the 22 participants remained in the LIB program at the final post-program follow-up. Participants demonstrated significant decreased waist circumference and elevated HDL cholesterol. Triglycerides manifested the highest percentage change without statistical significance. No significant change was observed in blood pressure or fasting blood glucose.

**Conclusions:**

LIB participants’ improvements in BMI, waist circumference, HDL cholesterol and triglycerides suggests type 2 diabetes prevention programs aimed at urban AI/ANs show significant potential for reducing the risk of developing type 2 diabetes among this underserved and high risk community. Qualitative data suggest the main challenge for type 2 diabetes prevention specific to this population is a need for improved community outreach strategies.

## 1. INTRODUCTION

Type 2 diabetes is a global public health crisis affecting all socioeconomic classes and all races [1]. Minorities such as American Indian/Alaska Natives (AI/ANs) suffer from a disproportionately high burden of type 2 diabetes [2]. AI/ANs have the highest age-adjusted prevalence and incidence of type 2 diabetes among all US racial and ethnic groups in spite of the fact that numerous programs funded by the US government have been conducted in AI/AN communities to prevent and treat type 2 diabetes since 1997 [3]. The causes of the disproportionately high prevalence of type 2 diabetes among AI/ANs beyond those associated with modifiable lifestyle and dietary factors remain unclear, although these race/ethnicity disparities may be accounted for by genetic predisposition and/or the effects of metabolic programming during early development—especially during pregnancy and nursing [4]. Although 60% of AI/ANs live in urban areas [5], Indian Health Service (IHS) and tribal health services are not as accessible to these populations relative to those living on reservations [6–8]. Previous research among AI/AN populations has been primarily conducted in federally recognized tribal communities; few studies have investigated the full extent of the problem of type 2 diabetes in the underserved US urban AI/AN population.

The Life in BALANCE (LIB) project was a community-based participatory research project piloting a translational study that modeled the Diabetes Prevention Program (DPP) intensive lifestyle coaching intervention among an urban AI/AN population. The purposes of the study were to: 1) describe the translational type 2 diabetes prevention program specifically modified for an urban AI/AN population; 2) utilize the results of the pilot project to inform larger future type 2 diabetes prevention programs targeting urban AI/AN populations through diet modification and increased physical activity.

## 2. RESEARCH DESIGN AND METHODS

The LIB project targeted urban AI/AN residents in Las Vegas, Nevada at increased risk for developing type 2 diabetes. Participants were recruited through community screenings, media announcements and referrals from other participants and/or project members. The inclusion criteria for participant eligibility included: being 21 years of age or older; AI/AN self-identification; a BMI ≥ 25 kg/m^2^; and a HbA1c level between 5.4% (36 mmol/mol) and 6.4% (46 mmol/mol) from a whole blood capillary blood sample. Participants with major illness or using medication known to interfere with glucose tolerance were excluded from this study. Qualified screened participants were informed about the LIB study and those who expressed an interest in and were able to meet the demands of participation in the LIB type 2 diabetes pilot prevention project received a 16-week type 2 diabetes prevention core curriculum and regular follow-up. The intensive interventions accentuated a nutritionally balanced portion control weight loss plan including: weight-loss curriculum, meal planning, fat gram and calorie counting, portion size, and food content education. Target measures for participants were directed towards caloric and fat gram profiles based upon individual goals. Core group sessions were primarily conducted at the Las Vegas Indian Center (a community-based information and resource center) and scheduled on a weekly basis in order to accommodate participant availability as much as possible. On a few occasions sessions took place at a different venue in order to emphasize an educational goal from the curriculum. These included restaurants and a fitness center to illustrate core lessons. LIB Native lifestyle coaches received centralized training that included: required reading, instructional videotapes, observation of trained personnel, and audio/video taped practice sessions and resource core review.

Participant demographic, lifestyle and dietary information was obtained before and after the completion of the LIB lifestyle core curriculum using a baseline survey and a post-participation survey. Participants fell into three groups, those who completed all 16 sessions of the LIB Program Curriculum, and who provided the baseline, post-curriculum and final measures and remained in contact after a period of self-maintenance (Completers, n = 12), those who completed all 16 session of the LIB Program Curriculum and provided the baseline and post-curriculum measures (Partial Completers, n = 3), and those who discontinued at various points in the program and only provided baseline measures (Non-completers, n = 7). Clinical measures included body weight, BMI, waist circumference, blood pressure, blood lipids, fasting blood glucose, and HbA1c, and were collected before and after the completion of the LIB lifestyle core curriculum and the end of post-program follow-up when possible. All clinical procedures were followed standardized protocols and performed by trained clinical specialists.

The SPSS statistical package (SPSS 19, IBM, Armonk, NY) was used for analysis of the quantitative data (clinical measures). Descriptive statistics was used to summarize the data. The Wilcoxon signed-rank test and the Fisher’s exact test were used to compare the differences of demographic information and baseline clinical measures between participants who completed the program and those who dropped out. The Friedman test was used to analyze the change of clinical measures across the three time points. Intention-to-treat analysis was used to analyze the influence of dropouts. Wilcoxon signed-rank test was conducted as post hoc analysis with a Bonferroni correction applied.

The LIB qualitative component used semi-structured one-on-one interviews to understand the experiences of 11 of the program participants who completed the core curriculum (n = 11 out of 15). Subjects who agreed to participate in the qualitative component ranged in age from 23 to 55 years with a mean of 39 years and included 9 women and 2 men. Interviews were transcribed and then coded and analyzed using Atlas.ti Qualitative Data Analysis software. A guideline of ten open-ended questions was used by the interviewer, but participants were encouraged to speak openly about their experiences. All interview comments were coded into condensed phrases (e.g., “My mom was fifty five, but my grandmas—both my grandmas—were in their eighties, so they were older but their quality of life over probably the last twenty years of their life wasn’t that great. And I thought, do I really want to be there?” was coded as “Worry because of family history”) and codes were then organized into non-overlapping categories (e.g., “Worry because of family history” was categorized under “Motivations for signing-up for the LIB Program”).

## 3. RESULTS

### 3.1. Quantitative Results

A total of 22 (age, 39.6 ± 10.4 years; BMI, 34.1 ± 6.3 kg/m^2^) subjects were enrolled in this pilot study, 10 of them (3 Partial Completers, 7 Non-completers) dropped out of the program before providing the final set of clinical measures (dropout rate: 45.5%). The demographic information and baseline clinical data are summarized in Table 1. Based on subjects’ characteristics at entry, there were no significant differences in demographic information and clinical measures between those participants who completed the program and those who dropped out of the program.

Among the clinical measures, body weight, BMI, waist circumference, HDL cholesterol, and total cholesterol to HDL ratio (TC/H), demonstrated statistically significant improvement after the LIB intensive lifestyle intervention (Table 2). Twelve core curriculum completers demonstrated a steady drop in weight (5.79%, p = 0.01), BMI (5.90%, p = 0.01), and waist circumference (4.34%, p = 0.01) across the three time points, but significant rise of HDL cholesterol (12.20%, p = 0.01) and drop of TC/H (6.78%, p = 0.01) occurred after the completion of the core curriculum.

Triglycerides showed the largest mean reduction (15.89%), but the change was not statistically significant because of a high variability. Systolic blood pressure decreased more than diastolic blood pressure. Fasting blood glucose and HbA1c showed very limited improvement after the program.

The results from the intention-to-treat analysis were highly consistent with those from the original analysis (Data not shown), indicating that positive findings did not result from removing the data of drop-outs. However, the results from the intention-to-treat analysis are not considered in the final results due to the risk of introducing bias and inflating type I error.

### 3.2. Qualitative Results

Eight thematic categories emerged as the most relevant to the aims of the qualitative component, and the most common sets of codes for each (those reported by the greatest number of participants) are listed in Table 3. Each thematic category is supplemented with a selected quote providing a richer description of participants’ experiences as reported during the interviews.

## 4. DISCUSSION

### 4.1. Quantitative Results

Weight and waist circumference are universal parameters in estimating general and abdominal obesity, respectively. Abnormal body fat distribution around the abdomen is highly associated with an increased risk for type 2 diabetes, even if the BMI falls within the normal range [9]. This is because the development of type 2 diabetes is influenced more by visceral fat tissue which is metabolically more active and produces more hormones and cytokines [10]. In the present study, both waist circumference and body weight (or BMI) showed a significant reduction at the completion of the core curriculum, although this trend slowed down in the follow-up period. The core curriculum was the most structured phase of the LIB lifestyle intervention involving culturally informed strategies to increased physical activity and decreased calorie and fat intake. All the participants were required to set individualized goals for weight loss and fat/calorie intake before the core curriculum and were encouraged to achieve these goals within 4 months. The early achievement of a significant reduction of waist circumference might be attributed to the presence of Native lifestyle coaches and an incentive package that included a free gym membership. With the guidance and supervision of the Native lifestyle coaches, as well as access to fitness resources, participants were more prone to follow the scheduled exercise and diet plan. However, after removing extrinsic motivations, a self-managed and self-monitored plan was employed to reinforce the acquired positive behavioral changes in the follow-up period. This may have contributed to the deceleration of the waist circumference and body weight (or BMI) reduction in the follow up period. No weight and waist circumference regain occurred at the end of the follow-up period, however, indicating that the core curriculum was effective in helping participants master weight maintenance skills. The effect of the LIB lifestyle intervention persisted at least 35 weeks.

HDL cholesterol and triglycerides are two typical lipoproteins in diabetic dyslipidemia, although their responses to lifestyle interventions may differ. A previously conducted 36-month clinical trial found that both diet alone and diet combined with exercise decreased triglyceride levels and increased HDL cholesterol values in participants with metabolic syndrome [11]. Two similar studies found that no change occurred in HDL cholesterol from baseline after 5-month and 6-month lifestyle intervention respectively, regardless of a significant reduction in waist circumference and triglyceride levels [12,13]. Results from the three studies suggest that triglycerides have a quick response to lifestyle intervention while HDL cholesterol demands a longer period to manifest observable positive change. In the present study, HDL cholesterol remained steady throughout the 16-week core curriculum, but a statistically significant elevation was observed during the follow-up period. No significant change was detected in triglycerides, but triglyceride values showed the highest percentage of decrease, which mainly occurred immediately following the core curriculum phase. These findings are in concordance with the assumption above, indicating that elevation of HDL cholesterol has a long lag time and a 16-week ongoing LIB lifestyle intervention is effective in increasing HDL cholesterol in the long term. Change in TC/H level was inversely consistent with that of HDL cholesterol because the variation in total cholesterol was very limited.

Many studies have indicated that hypertension can be relieved through lifestyle intervention, especially in decreasing systolic blood pressure [11,14]. A smaller decrease was observed in diastolic blood pressure compared with systolic blood pressure in the present study, but neither was statistically significant. One possible explanation is that the average baseline blood pressure among our participants was within normal range (systolic BP: 119.19 ± 13.19 mmHg; diastolic BP: 76.54 ± 9.74 mmHg), which might have limited the effect of the LIB lifestyle intervention in alleviating hypertension.

Hyperglycemia is one of the primary symptoms in individuals with prediabetes and type 2 diabetes. It is also the most intractable target in type 2 diabetes prevention programs. Diet and exercise are widely perceived to be beneficial for glycemic control. However, conflicting results on the effects of lifestyle intervention in improving hyperglycemia are common in clinical trials. In the present study, fasting blood glucose was not affected by the LIB lifestyle intervention. In addition, no sign of improvement in long-term glycemic control was observed, which was evidenced by stable HbA1c levels. This finding is consistent with a recent study in an overweight and obese female Canadian population, reporting that fasting blood glucose was not affected by diet and exercise regardless of significant weight loss [14].

Moderate weight loss is beneficial in improving insulin resistance [15]. Previous studies have shown that a 10% weight loss lowers blood glucose and improves cardiovascular risk factors. It is possible that an average weight loss of less than 10% is not sufficient to trigger a detectable reduction of blood glucose. On the other hand, considerable evidence showed that an isocaloric diet, low in fat and enriched in carbohydrates, will exacerbate insulin resistance. Replacing saturated fat with carbohydrates may increase daily blood glucose level because it requires more insulin to maintain glucose homoeostasis, which is demanding in individuals with insulin resistance [15]. The LIB core curriculum emphasizes reducing fat and total calorie intake without a structured guide for dietary macronutrient content. High levels of carbohydrate intake might have counteracted the effect of weight loss in improving insulin resistance and lowering blood glucose. The addition of a structured dietary macronutrient content guide in the LIB core curriculum may be effective in achieving more weight loss and an observable fasting blood glucose reduction.

### 4.2. Qualitative Results

The aim of the qualitative research component was to understand the specific challenges and successes of participants who completed this study, and not captured by the clinical measures, in order to inform the design of future type 2 diabetes prevention programs geared toward urban AI/ANs. Contrary to one of the major assumptions motivating this study, the most commonly reported barriers to adherence with lifestyle goals for those who finished the LIB Program—family, work, social obligations, time, money—are not unique to urban American Indians. Worldwide meta-analyses of compliance issues in type 2 diabetes intervention programs report strikingly similar issues [16,17]. For example, one of the most widely reported factors that helped participants in this study adhere to dietary goals—“Knowledge about nutrition facts”—has been shown in numerous studies to be a critical factor for improving health outcomes in pre-diabetic subjects worldwide [18,19]. In the present study, the only instances when participants mentioned anything overtly specific to American Indians in their comments was in the context of making suggestions to the interviewer that the project team find better ways to target this group because of knowing other American Indians (friends, family, co-workers) in need of and expressing an interest in being involved with a program similar to this one:

Interviewer: *Is there anything else about your experiences in the Life in Balance Program that you would like to share that could help programs like this in the future*?

*Mm... I think more—you need to get it out there more to the natives... more advertising. I guess you could say reach out more than to just basic—I don’t know—you know*, *get out there in the communities and look for the people who do need help and stuff. I’m not sure how many natives live here. It would be nice to get them all together*, *you know*, *and teach them and stuff*… *more outreach*—26-year-old Navajo female.

Although the challenges of lifestyle goal adherence among urban AI/ANs in the current study appear to be very similar to those of other prediabetics from a broad range of ethnic/racial and socioeconomic backgrounds, the value of developing a type 2 diabetes prevention program aimed specifically at urban AI/ANs, and targeting program recruitment and participation based on AI/AN identity, was nevertheless an important factor regarding interest and participation in the LIB program.

… *the way I found out* [*about the program*] *was one of my friends that works for the-she used to work for the Indian Education Center. She’s the one that emailed me and told me about it and I thought wow I mean you can’t even-going home and trying to get in something like this and learning about diabetes*, *you only learn when they have the health fairs*, *you can go to the hospital and find out but some are too much in a hurry*… *they just don’t realize*, *you know*, *how important it is*—55-year-old Navajo female.

Interviewer: *What did you find interesting about the Life in Balance Program*?

… *well*, *um*, *the program itself and the goal of the program for Native Americans. This is a huge issue in my family. It’s kind of always on my mind as I get older and um I really think it should be directed towards youth and younger generations*, *just because it’s such a huge problem in Native American communities*—37-year-old Choctaw female.

## 5. CONCLUSIONS

Some limitations of the study described here should be noted. First, the pilot study was hampered by a small sample size. Even the intention-to-treat analysis indicates that the positive changes may be attributable to the lifestyle intervention itself, but large variability resulting from the small sample size might have masked some significant results. Second, the last follow-up visit was completed at either 8 months or 12 months after the first clinical measure. Theoretically, acquired positive behaviors gradually weaken over time without proper reinforcement. Participants who had an early follow-up visit might behave better than those who had a late follow-up visit, which might have influenced the last round of clinical measures. Third, due to the nature of secondary analysis, some important confounding factors cannot be addressed in this study. For example, social support is crucial in shaping and consolidating individuals’ positive behaviors. Information like marital status was not collected in the LIB pilot study, which might also have helped explain the high dropout rate. Information on lifestyle choices like smoking and drinking was not collected, which also plays a role in increasing metabolic risk related to type 2 diabetes, and which might have attenuated the effects of the LIB program.

According to the qualitative information gleaned from interviews with study participants, the main challenge for type 2 diabetes prevention interventions specific to urban American Indians may be locating and reaching this particular population; a population, in spite of suffering from a greater disease burden, has both health challenges and health aspirations that are indistinguishable from other middle class working Americans. It is clear from this study, through the analysis of interview data and the experiences of the research team during the planning stages of the project, that the need for improved recruitment strategies should be a primary focus in the design of future type 2 diabetes prevention programs among urban American Indian populations.

Importantly, both this issue and the majority of other issues describing that are not specific to urban American Indians, and can theoretically be at least partially ameliorated by incorporating more technologically savvy tools into the program. For instance, taking advantage of existing or creating customized smart phone applications to keep track of diet and exercise goals may greatly reduce both participant and researcher burden, a potential that has yet to be fully realized in the health sciences in general [19]. The use of online social networking tools (e.g., Facebook, MySpace, etc.) as a recruitment strategy offers many advantages for both participants and researchers over the use of email lists provided by community centers or the “word-of-mouth” techniques that were predominantly used in this study. In addition, many of the more individualized problems reported by participants regarding LIB group dynamics and lifestyle coach teaching styles could also be addressed by an online social networking strategy, particularly one similar in function to popular dating websites (e.g., Match.com, eHarmony) which narrow down prospects according to shared answers to relevant questions. A group-based type 2 diabetes intervention program might include questions such as: “Do you work better with a big group or a small group?”; “Are you more successful when you work out on your own or with friends?”; “Do you work better with a more involved lifestyle coach or a more hands-off approach?”; “What is your tribal affiliation?”. Finally, technological improvements in personalized medical equipment, such as commercially available HbA1c self-check systems, could be offered as joining incentives, simultaneously providing participants and researchers with immediate results progress.

In summary, the results indicate that the LIB program reduced the risk for type 2 diabetes and its complications in urban AI/ANs living in Las Vegas and at an increased risk. The current LIB core curriculum is effective in decreasing weight, waist circumference and elevating HDL cholesterol level. Qualitative data from informal interviews suggest that the main challenge for type 2 diabetes prevention research specific to this population is a need for improved community outreach strategies because, in spite of suffering from a disproportionately higher type 2 diabetes burden, urban AI/ANs in this study faced challenges not different from other working class groups living in urban areas. A more technologically savvy program design and recruitment strategy offer many promising and creative solutions to many of these challenges, and may extend the perceived benefit of participating in a type 2 diabetes prevention program with other AI/AN participants. Further research is needed to investigate the effect of an optimized core curriculum in strengthening the efficacy of the LIB program among AI/ANs living in urban environments.

## Figures and Tables

**Table 1:** Demographic characteristics and baseline clinical measures of the study population.

Variables	Participants who completed the program (n = 12)	Participants who dropped out (n = 10)	p-value
Female (%)	83.33	70.00	0.624
Age (years)	38.58 ± 9.55	40.70 ± 11.81	0.923
Education (%)			0.074
Without bachelor degree	50.00	90.00	
Bachelor degree or higher	50.00	10.00	
Employment (%)			0.293
Unemployed	8.33	30.00	
Employed	91.67	70.00	
Household income (%)			1.000
<$ 50,000	66.70	70.00	
≥$ 50,000	33.30	30.00	
Weight (kg)	96.67 ± 12.50	94.63 ± 28.64	0.381
Waist circumference (cm)	110.89 ± 11.40	113.00 ± 21.64	0.974
Triglycerides (mg/dL)	189.92 ± 109.41	152.10 ± 47.10	0.539
HDL cholesterol (mg/dL)	49.91 ± 15.42	48.00 ± 7.17	0.863
Fasting blood glucose (mg/dL)	107.17 ± 10.26	107.50 ± 6.40	0.923
Systolic BP (mmHg)	121.03 ± 14.49	116.99 ± 11.81	0.628
Diastolic BP (mmHg)	78.07 ± 11.08	74.70 ± 8.04	0.418

1) Values shown are mean values ± standard deviation (SD); 2) Results are based on the Wilcoxon signed-rank test and the Fisher’s exact test.

**Table 2:** Comparison of clinical measures over three time periods (n = 12).

Variables	Baseline (1st)	Completed curriculum (2nd)	Follow up (3rd)	p-value	Change
Weight (kg)	96.67 ± 12.50	93.53 ± 12.44	91.07 ± 10.37	0.010*	−5.79%
Waist circumference (cm)	110.89 ± 11.40	108.20 ± 11.66	106.08 ± 11.09	0.010*	−4.34%
Triglycerides (mg/dL)	189.92 ± 109.41	158.42 ± 75.30	159.75 ± 72.14	0.717	−15.89%
HDL cholesterol (mg/dL)	49.91 ± 15.42	48.64 ± 15.00	56.36 ± 14.62	0.007*	+12.92%
Fasting glucose (mg/dL)	107.17 ± 10.26	105.17 ± 7.94	106.75 ± 4.90	0.502	−0.39%
Systolic BP (mmHg)	121.03 ± 14.49	117.00 ± 10.26	113.72 ± 10.25	0.338	−6.04%
Diastolic BP (mmHg)	78.07 ± 11.08	78.71 ± 12.02	76.55 ± 11.82	0.779	−1.95%

Post Hoc Tests			
Variables	1st vs. 2nd	1st vs. 3rd	2nd vs. 3rd
Weight (kg)	1st > 2nd, p = 0.003	1st > 3rd, p = 0.012	2nd = 3rd, p = 0.347
Waist circumference (cm)	1st > 2nd, p = 0.016	1st = 3rd, p = 0.050	2nd = 3rd, p = 0.230
HDL cholesterol (mg/dL)	1st = 2nd, p = 0.212	1st = 3rd, p = 0.045	2nd < 3rd, p = 0.004

1) 1st refers to the first clinical measure before the LIB program; 2nd refers to the second clinical measure at the completion of the LIB core curriculum; 3rd refers to the third clinical measure at the end of follow-up; 2) This procedure only included participants completing all three clinical measurements (n = 12); 3) Values shown are mean values ± standard deviation (SD); 4) Results are based on the Friedman test; 5) Post hoc results are based on the Wilcoxon signed-rank test with a Bonferroni correction (p < 0.0167); insignificant results are reported as equality, and no post hoc analysis was done; 6)

* Indicates significant at 0.05.

**Table 3:** Participant experiences with LIB program.

Thematic categories	Interview quotes
Motivations for signing-up for the LIB Program	
Needing a life change Fear/worry about state of health because of family history Inspiration from hearing success stories	My mom was fifty five, but my grandmas were in their eighties, so they were older but their quality of life over probably the last twenty years wasn’t that great. And I thought, do I really want to be there? Do I want to not be able to, you know, get up and walk to the car? Do I want to have someone pushing me around? Because I’ve been to that gym now, and I see people who are like in their—they’ve got to be in like their sixties maybe seventies—and I’m like, that’s what I want to be like. —43-year-old Oglala Sioux female
Motivations for finishing the Life in Balance Program	
Feeling accountability to self, family, lifestyle coach, group Seeing/feeling immediate positive results	… if you have loved ones, children, then you don’t want to die early because you have to be there for them and that’s what my kids were—my motivators. When I found out how bad my blood was and I was like borderline diabetic… it was really bad. I was really scared for myself and I think right away after that I went and got life insurance because I was so scared. —34-year-old Choctaw/Creek female
Factors that affected overall participation	
Time constraints Work schedules/situations Household dynamics Support system Social obligations	Since I’ve only lived in Vegas for probably just over a year now a lot of people found out that I did move here so they wanted to come and some of them ended up staying with me. So they always wanted to go eat out or they wanted to go have a drink or something… that kind of interfered with me actually watching what I ate and what I put in my body. —23-year-old Navajo female
Factors that helped with making diet changes	
Knowledge about nutrition facts Planning ahead Seeing/feeling positive results Writing things down/keeping track of progress Finding what works and doesn’t work on an individual basis Cognitive strategies Getting the household involved	And the actual reduction of the blood sugar-glucose—HbA1c… it was more gratifying to actually see it on paper versus knowing that if I got it down it would benefit my health. But actually doing it and seeing it was very gratifying. —44-year-old Oglala Sioux male
Difficulties with making diet changes	
Work schedules/situations Household dynamics Time constraints Financial constraints Cognitive struggles Picking up other unhealthy habits Social obligations	… if you are not cooking for someone else and eating alone everyday it’s hard. And the situation that we were in yesterday is a little bit different than others, but with a combined household… and I’m trying to put weight on my son, and take weight off of me, and just making separate meals, or getting everybody to eat something—at least cooking something—that everyone will like that is also healthy… that’s challenging. —37-year-old Choctaw female
Factors that helped with making exercise changes	
Physical setting Social setting Seeing immediate results Cognitive strategies	I think maybe it’s just that momentum when you walk in [to the gym], you kind of like get in that zone, and then, you know, they have everything there and just the environment, the atmosphere, everybody’s there working out and just getting in there and doing, but, more importantly, sticking to it. —44-year-old Oglala Sioux male
Difficulties with making exercise changes	
Time constraints Work schedule/situations Household dynamics Social support Long-term maintenance Cognitive struggles	The other group [I started out in] was all women, um, I think we were all single… you know it almost had a competitive edge to it. The larger group we laughed, at each other if we needed to, but I think there was also a lot of support there and a lot of honesty. I think that it gave us an opportunity to learn from each other and see what our struggles were. Because I think that we all had different kinds of struggles, you know? —48-year-old Cherokee female
Suggestions for improving the LIB Program	
Better materials, additional information Additional/alternative ways of relaying information Better community outreach strategies for American Indians and youth	I’m not a kind of classroom person. I think that people—if you actually take them out there and do it with them, the exercise… learn different exercises together, go out on trails and go out on hikes, just make a more interactive and just-just real world examples. —37-year-old Choctaw female
